# Death education for Palliative care: a european project for University students

**DOI:** 10.1186/s12904-023-01169-6

**Published:** 2023-04-21

**Authors:** Ines Testoni, Lucia Ronconi, Hod Orkibi, Gianmarco Biancalani, Melania Raccichini, Luca Franchini, Shoshi Keisari, Mihaela Bucuta, Krzysztof Cieplinski, Michael Wieser, Silvia Varani

**Affiliations:** 1 Department of Philosophy, Sociology, Education and Applied Psychology (FISPPA), University of Padova, Via Venezia 14, Padua, 35131 Italy; 2 Drama & Health Science Lab, and the Emili Sagol Creative Arts Therapies Research Center, Faculty of Social Welfare and Health Sciences, University of Haifa, Haifa, Israel; 3 IT and Statistical Services, Multifunctional Pole of Psychology, University of Padova, Padova, Italy; 4 ANT Foundation, Bologna, Italy; 5 Department of Journalism, Public Relations, Sociology and Psychology, Lucian Blaga University of Sibiu, Sibiu, Romania; 6 Department of Psychotherapy and Health Psychology, The John Paul II Catholic University of Lublin, Lublin, Poland; 7 Department of Psychology, Alpen-Adria-Universität Klagenfurt, Klagenfurt, Austria

**Keywords:** Palliative psychology, University death education, Death competence, Creative self efficacy, Palliative care career commitment

## Abstract

**Background:**

The need to spread the culture of palliative care and to train health care professionals from undergraduate courses is recognised internationally. The article presents the outcomes of a project devoted to palliative care training in university courses in four countries.

**Aims:**

This article considered the outcomes of a course designed for university students who had the potential to work in a palliative care team. The main aim was to check the efficacy of the course and the motivation to work in palliative care settings, considering the impact of fear and representations of death.

**Methods:**

The project presented the essential contents related to palliative care, using psychodramatic and photo-voice techniques. Longitudinal measurements were taken using a quantitative method design to detect changes among the students involved. The project involved 341 students at the first administration of the survey consisted of a protocol composed of standardized questionnaires in five countries (Austria, Israel, Italy, Poland and Romania), of whom 276 completed the pre- and post-surveys—165 of them in the experimental group and 111, in the control group.

**Results:**

The experience showed that it is possible to address death-related issues seriously and competently without necessarily causing discomfort and despondency in students. The results of the changes over time in the experimental and control groups highlight how the view of death as annihilation is correlated with the fear of death and the need for avoidance of thoughts concerning dying. The main result is that competence in palliative care facilitates familiarisation with issues of death and dying, as well as the ability to work in this area, thereby enhancing interpersonal skills.

**Conclusion:**

The project showed that it is possible to implement death education on palliative care topics in undergraduate courses to increase motivation to work in this field.

## Introduction

Since the second half of the 20th century, medical research has been growing and has considerably improved the quality and duration of life in Western countries [1]. This achievement also involves those suffering from serious illnesses. In keeping with the perspective that values quality of life at every stage of the evolution of an individual’s health conditions [2], palliative care (PC) should be offered simultaneously with all other medical treatments or, at least, during the terminal phase [3]. In line with the conception of health of the World Health Organization (WHO) as a harmony between the somatic, psychological and social dimensions [4, 5], PC aims to relieve patients’ suffering and increase their quality of life as well as that of their families, thanks to the specific knowledge and skills of the healthcare professionals involved [6]. Although the WHO [7] highlighted PC as a human right, most people do not yet take advantage of this treatment. The reasons why access to PC is not yet universal are manifold and depend on the different levels of wealth in different geographical areas, local regulations and culture, and discrimination of minorities. One of the cultural reasons that is common to almost all countries, including the most advanced Western countries, is a general ignorance about PC. Indeed, knowledge of PC by health professionals and society is still lacking [3] because of the general avoidance of the cognition of death in everyday life [8, 9]. Indeed, the scarcity of understanding and of social discussion on such issues, even in health-related professional degree courses, has negatively impacted PC development and its implementation [10, 11].

Healthcare professionals’ lack of knowledge of PC is partially ascribable to university degree courses that still inadequately prepare future healthcare professionals with respect to PC and the management of death and dying [12]. Ignorance of the availability now of appropriate PC programmes for better management of these conditions makes the experience of dying even more painful for patients and their families. The cost of incompetence in this area jeopardises the quality of care in the most serious stages of illness, as it prevents the building of an effective alliance with patients for their treatment or for their making of the best end-of-life choices [12]. Death education (DeEd) is an enlightening pathway for potential healthcare professionals to meet the formative demands of PC and to acquire cognitive strategies that improve their relational and communication skills because through it, they learn the different forms of dying and the meanings attributed to death [13–18] and then how to manage death in different contexts and situations [19, 20].

## Death education for palliative care

PC competencies beyond the management of pain and other symptoms include relational and communication skills for the construction of a good therapeutic alliance with patients and their family members and for the provision of good psychosocial, spiritual and bereavement support [21]. DeEd focused on PC is particularly appropriate in university degree courses for potential healthcare professionals [22] because it prepares them to manage fear of death and its negative impact on communication and relational issues [13].

Indeed, PC begins with clear communication between healthcare professionals and patients and their families on the goals of the care, especially when active treatments may be more oppressive and onerous than beneficial [23]. Literature shows both the benefits and the lack of early end-of-life care discussions with patients with life-threatening illnesses and with their families [24]. The effectiveness of healthcare professionals in the use of specific communication skills during such demanding contexts decreases the anxiety and depression of the patients and their families and improves their satisfaction with the treatment [3, 25]. The negotiation of realistic and manageable goals that facilitate decision-making on treatments and advanced directives is possible only if the death competence of healthcare professionals is adequate and if their communication abilities are guaranteed [3]. Even though communicating with patients should be a core skill of all healthcare professionals, literature shows that during the treatment, they fail to consider their patients’ points of view, goals of care or treatment preferences [26].

Furthermore, shared care planning, which is the process of facilitating communication between patients and healthcare professionals to support long-term care in facing death, is based on efficacious communication with related management of emotional states [27], especially of the fear of death [28]. If fear of death and death anxiety are effectively held at bay, a successful therapeutic alliance between patients and their health teams can be achieved through increased transparency and provision of timely information, shared decision making and patient engagement, which will result in higher quality of care [29, 30]. DeEd may be an adequate means of improving this competency because it offers healthcare professionals integrated expertise in managing advanced treatment directives with end-of-life choices, PC and bereavement elaboration [3, 31]. Many specific courses pivoted on cognitive, behavioural and affective factors and aimed to improve communication skills ameliorated the relational abilities in health-operator–patient relationships [32, 33], by helping the participants to become aware of their representations of death, thereby reducing their anxiety and distress [34].

Doka [31] also described the positive effects of activities of DeEd courses: the resulting psychological competencies are significant because they improve and support all the psychological skills necessary for facing the fear of death.

## The project background and objectives

In Europe, the Death Education for Palliative Psychology (DE4PP) Erasmus Plus project developed curricula for university training paths dedicated to PC from a psychological perspective—understood as intersectional knowledge for all health professions involved in PC—utilising psychodrama and creative arts techniques [18]. DE4PP was based on the idea that psychology can provide important skills for relationships and communication with patients and their family members after the inauspicious prognosis and for the management of PC. The project involved five universities (University of Padova in Italy, University of Klagenfurt in Austria, University of Haifa in Israel, John Paul II Catholic University of Lublin in Poland and Lucian Blaga University of Sibiu in Romania) and the Italian Onlus Assistenza Nazionale Tumori (ANT; National Tumour Assistance), which provides medical assistance to often terminally ill cancer patients. The aim of the project was to train students from European universities, who might take part in a multidisciplinary PC team (physicians, nurses, psychologists and social workers) in the future, on fundamental death competence, the theoretical basis of PC and psychological strategies for good communication and relationships with dying patients and their families.

The present study aims to evaluate the impact of a PC DeEd course on psychology students. Specifically, to assess whether the course could provide the necessary tools to familiarize them with death-related issues so as to (a) manage possible anxiety or attitudes of rejection derived from the topic of death; (b) stimulate an interest in these issues for a future career; (c) evaluate whether creative arts therapies could be a useful tool to be employed of PC DeEd interventions.

## Methods

### DE4PP project intervention

The groups of students from each DE4PP partner country were enrolled in university degree programmes in psychology. The courses were divided into two parts: face to face and online on a specially created Moodle platform. For the online part, the course consisted of nine modules, each of which comprised three tasks: viewing of a 20-min video lesson, review of the PDF document of the video lesson slides and answering of the questionnaire that evaluated the students’ understanding of the video lesson. The students could not proceed to the next module without completing the three tasks in the previous module. To give the students access to the online course, their email addresses were collected, and they were enrolled in the Moodle platform, where they could follow the lessons. The first five modules focused on explaining death education and palliative psychology: PC, communication skills, relational skills, advanced care planning and strategies to provide psychological support to patients. To improve the students’ relational abilities, the further four modules focused on the use of art therapies, particularly psychodrama, photovoice and intermodal arts therapies. The psychodramatic techniques were particularly efficacious because the guided role playing permitted, on the one hand, emphasis on and familiarisation with grieving and dying persons before meeting them, and on the other hand, practice of the relational and communicative competencies [35–38]. At the end of each of the last four modules and before starting the next one, the students took part in a 3 h face-to-face or online training workshop on the use of the methodologies illustrated in the online lessons. Italy, however, has taken a partially different course from the other countries to better understand whether it is possible to change the representation of death as annihilation, to reduce the fear of death and avoidance. For this reason, 4 h were devoted to topics related to religions, spirituality and the discussion of representations of death that influence attitudes and behaviour, quoting Terror Management Theory (TMT) studies [39].

### Participants

A total of 341 psychology students participated in this study at the first administration of the survey. Of these, 276 completed the pre- and post-surveys, 165 of them in the experimental group and 111 in the control group. The numbers of students in the experimental and control groups per country were 64 and 56 in Italy, 30 and 20 in Austria, 22 and 10 in Israel; 26 and 13 in Poland and 23 and 12 in Romania. The additional characteristics of the participants are shown in Table 1.

The experimental group students were recruited through convenience sampling. They were volunteers from the curricular courses of the project (one from each state). They were asked who wanted to participate in this free online course on the topics of death education and palliative psychology. The students in the control group were selected through convenience sampling, either by asking students who were not in the experimental group to join the control group or by asking students in the experimental group for the names of students whom the researchers could contact to be part of the control group. All the students who took part in the study are psychology students from universities where the professors involved in the DE4PP project teach psychology.

### Data collection and measures

Data were collected through an online survey, developed on Qualtrics, that could be accomplished in about 20 min. The survey consisted of a protocol composed of a series of standardized questionnaires. The protocol in its overall composition has never been used before, while instead the individual questionnaires had precisely been validated and used in previous studies. The validated version in each country’s reference language was used for all questionnaires; if not present, the questionnaires were translated by back translation. The students in the experimental group answered the protocol twice, before and after finishing the course. They found the protocol link on the Moodle platform (there was a link at the start of the course and one at the end). The students in the control group also answered the protocol. They received the protocol link by email from their professor exactly when the experimental group was taking the survey before the course and exactly when the experimental group was taking the survey after the course. The standardized questionnaires of which the protocol was composed are as follows.

The Testoni Death Representation Scale (TDRS) [40] is a six-item five-point Likert scale (from 1 = strongly disagree to 5 = strongly agree) that investigates the representations of death as a passage or annihilation. The original scale was validated in the Italian language, an example of its items is “Death is only a passage. After I die, I will continue to exist and will remember this life’s experiences.”

The Death Attitude Profile – Revised (DAPR) [41] is a 32-item five-point Likert scale (from 1 = strongly disagree to 5 = strongly agree) that investigates five attitudes towards death: fear of death, death avoidance, neutral acceptance, approach acceptance and escape acceptance. An example of its items is “Death is no doubt a grim experience.”

The Career Commitment Scale (CCS) [42] is an eight-item five-point Likert scale (from 1 = strongly disagree to 5 = strongly agree) that measures and predicts professional commitment and vocation, specifically for end-of-life care. An example of its item is “I definitely want a career for myself in end-of-life care.”

The Creative Self-Efficacy Scale (CSE) [43] is a five-item five-point Likert scale (1 = not true, 5 = very true) that measures the self-judgment of one’s imaginative ability and perceived competence in generating novel and adaptive ideas, solutions and behaviours. It was specifically modified for end-of-life care and arts therapies. An example of an item is “I am good at coming up with new ideas for arts-based end-of-life care.”

The Frommelt Attitude Toward Care of the Dying Scale – Form B (FATCOD) [44] is a 30-item five-point Likert scale (from 1 = strongly disagree to 5 = strongly agree) that measures the attitudes of medical and psychological personnel regarding the care of dying patients. An example of its item is “Giving care to the dying person is a worthwhile experience.”

The Compassion Scale (CS) [45] is a 16-item five-point Likert scale (from 1 = strongly disagree to 5 = strongly agree) that measures the psychological construct of compassion, understood as kindness, a sense of humanity, mindfulness and attention to the suffering of others. In this study, only two subscales were used: kindness (four items) and indifference (four items). An example of its items is “I am unconcerned with other people’s problems.”

### Statistical analysis

First, we examined the differences between the groups in terms of their demographic variables (i.e., country, age, gender, religion, religious level and year of master’s degree completion), previous experiences (as formal caregiver to end-of-life clients and as having lost someone close to them in the last two years) and baseline scores for the study variables. Second, we performed a 2 (time: Time1 and Time2) x 2 (group: experimental and control) x 5 (country: Italy, Austria, Romania, Israel and Poland) ANOVA to evaluate the change over time for each examined construct in the two groups and in each country. We also analysed the time effect inside each group via a t test and Cohen’s d as a measure of the effect size (d = 0.20, small effect; d = 0.50, medium effect; and d = 0.80, strong effect). Finally, we computed the change scores as differences from Time1 to Time2 in the experimental group, and we summarised the variability of these new variables through exploratory factor analysis, with the principal axis method for factor extraction and the oblimin method for factor rotation. Hierarchical regression analysis of the new components was performed, adding predictors in three steps: the control variables in the first step, the target variables at Time 1 in the second step and the significant interaction of the target variables at Time 1 with the moderator in the last step. We fitted several models, including as moderators in turn each target variable at Time1.

## Results

The two groups were similar in terms of all the demographic variables (Table 1) except for gender (chi-square = 3.88, df = 1, p = .049) and the religious level (t = -5.41, df = 274, p < .001). There were fewer female students in the experimental group, and they were less religious than the students in the control group. Moreover, only three study variables showed differences in their baseline scores between the two groups: the career commitment (t = 4.02, df = 274, p < .001), the creative self-efficacy (t = 2.20, df = 274, p = .028) and the attitude toward care of the dying (t = 3.34, df = 274, p < .001). The students in the experimental group had higher scores than the students in the control group.

**Table 1:** Descriptive Statistics for Demographic and Study Variables at Time 1 by Group

Variable		Experimental Group (N = 165)	Control Group (n = 111)	Group difference p-value
Demographic variables				
Country:				0.351
	Italy	64 (39%)	56 (51%)	
	Austria	30 (18%)	20 (18%)	
	Romania	23 (14%)	12 (11%)	
	Israel	22 (13%)	10 (9%)	
	Poland	26 (16%)	13 (12%)	
Age:		20–56; 27.16 (7.05)	21–51; 26.91 (7.39)	0.779
Gender:				0.049*
	Female	140 (85%)	102 (93%)	
	Male	25 (15%)	8 (7%)	
Religion:				0.635
	Christian	91 (55%)	60 (54%)	
	Jew	19 (12%)	9 (8%)	
	Moslem	0 (0%)	1 (1%)	
	Other	1 (1%)	1 (1%)	
	None	54 (33%)	40 (36%)	
Religious level		1–4; 2.36 (0.92)	1–4; 2.95 (0.85)	< 0.001*
Formal caregiver to end-of-life clients:				0.141
	No	154 (93%)	108 (97%)	
	Yes	11 (7%)	3 (3%)	
Lost someone close to you in the last two years:				0.471
	No	103 (62%)	74 (67%)	
	Yes	62 (38%)	37 (33%)	
Year of master’s degree:				0.821
	1st (or 4th in Poland survey)	90 (55%)	62 (56%)	
	2nd (or 5th in Poland survey)	60 (36%)	37 (33%)	
	3rd	15 (9%)	12 (11%)	
Study variables				
TDRS Total		7–30; 19.30 (5.88)	6–30; 18.80 (5.89)	0.488
DAPR Fear of Death		1–5; 3.06 (0.88)	1–5; 3.12 (0.87)	0.631
DAPR Death Avoidance		1–5; 2.31 (0.93)	1–5; 2.48 (0.96)	0.131
DAPR Neutral Acceptance		2–5; 4.02 (0.53)	2–5; 4.04 (0.57)	0.967
DAPR Approach Acceptance		1–5; 2.55 (0.97)	1–5; 2.54 (0.94)	0.905
DAPR Escape Acceptance		1–5; 2.72 (0.85)	1–5; 2.64 (0.82)	0.414
CCS Total		5–25; 13.19 (3.69)	5–25; 11.43 (3.37)	< 0.001*
CSE Total		5–25; 13.72 (4.59)	5–24; 12.45 (4.81)	0.028*
CS Kindness		11–20; 16.93 (2.04)	8–20; 16.91 (2.05)	0.926
CS Indifference		4–15; 7.33 (2.11)	4–14; 7.39 (2.18)	0.837
FATCOD Total		3–5; 3.91 (0.31)	3–5; 3.78 (0.35)	0.001*

The values reported in the table are the Range, Mean, (Standard Deviation) for continuous variables and frequency, (percentage) for nominal variables. The last column show the group difference p-value computed using the t test for continuous variables and the Chi-square test for nominal variables - *p < .05.

TDRS = Testoni Death Representation Scale; DAPR = Death Attitude Profile – Revised; CCS = Career Commitment Scale; CSE = Creative Self-Efficacy Scale; CS = Compassion Scale; FATCOD = Frommelt Attitude Toward Care of the Dying Scale.

### Changes over time in the experimental and control groups

The results of 2 (time: Time1 and Time2) x 2 (group: experimental and control) x 5 (country: Italy, Austria, Romania, Israel and Poland) ANOVA showed a significant time for group interaction for the following constructs: the death representation (F = 8.67, df = 1;266, p = .004), the fear of death (F = 10.00, df = 1;266, p = .002); the avoidance of death (F = 6.12, df = 1;266, p = .014), the neutral acceptance of death (F = 6.06, df = 1;266, p = .014), the career commitment (F = 14.89, df = 1;266, p < .001), the creative self-efficacy (F = 28.66, df = 1;266, p < .001); the kindness (F = 4.01, df = 1;266, p = .046) and the attitude toward care of the dying (F = 16.23, df = 1;266, p < .001). Moreover, a significant time for group for country interaction was found only for the death representation (F = 3.38, df = 4;266, p = .010), indicating similar changes over time between the experimental and control groups in each country. No difference over time was found for the acceptance of death as approach, the acceptance of death as escape and for the indifference. The ANOVA results also showed a significant main effect of the country for all the variables except for the career commitment and the attitude toward care of the dying. In particular, the Polish students had a lower incidence of annihilation as death representation than the students of the other countries. The Italian students expressed greater fear of death and avoidance of death, and the students of the other countries manifested more of the other death attitudes. The Italian and Austrian students were less creative than the students of the other countries. Finally, the Austrian and Israeli students showed less compassion (i.e., less kindness and greater indifference) than the other students.

To understand the time x group interaction, we examined the change over time in each group (see Table 2). Only the students in the experimental group showed a moderate to strong time effect on the death representation, the fear of death, the avoidance of death, the career commitment, the creative self-efficacy and the attitude toward care of the dying. In particular, they showed a lower incidence of annihilation as death representation, fear of death and death avoidance, and they demonstrated increased career commitment, creative self-efficacy and care for the dying. Moreover, only the students in the experimental group showed a small time effect on neutral acceptance of death, with only an increment of their baseline score after the course, and only the students in the control group showed a small time effect on kindness, with only an increment of their baseline score from Time1 to Time2.

**Table 2:** Change over time examination in experimental and control group

Variable	Experimental Group (N = 165)				Control Group (N = 111)			
	Mean difference	Standard Error	t	Cohen’s d	Mean difference	Standard Error	t	Cohen’s d
TDRS Total	1.21	0.29	4.13***	0.64	-0.25	0.40	-0.63	-0.12
DAPR Fear of Death	0.25	0.05	5.52***	0.86	0.00	0.06	0.04	0.01
DAPR Death Avoidance	0.21	0.05	4.14***	0.65	0.00	0.07	-0.06	-0.01
DAPR Neutral Acceptance	-0.09	0.04	-2.42*	-0.38	0.07	0.05	1.32	0.25
DAPR Approach Acceptance	-0.03	0.12	-0.25	-0.04	-0.01	0.12	-0.08	-0.02
DAPR Escape Acceptance	0.14	0.11	1.27	0.20	0.02	0.12	0.17	0.03
CCS Total	-1.29	0.20	-6.37***	-0.99	0.03	0.28	0.11	0.02
CSE Total	-3.01	0.34	-8.73***	-1.36	0.12	0.47	0.25	0.05
CS Kindness	0.01	0.14	0.08	0.01	0.50	0.20	2.53*	0.48
CS Indifference	0.16	0.17	0.94	0.15	-0.04	0.24	-0.17	-0.03
FATCOD Total	-0.13	0.02	-6.43***	-1.00	0.01	0.03	0.25	0.05

The mean difference is computed as difference of the score at Time 2 from the score at Time 1.

*p < .05; **p < .01; ***p < .001.

TDRS = Testoni Death Representation Scale; DAPR = Death Attitude Profile – Revised; CCS = Career Commitment Scale; CSE = Creative Self-Efficacy Scale; CS = Compassion Scale; FATCOD = Frommelt Attitude Toward Care of the Dying Scale.

### Exploration of changes in the scores of the experimental group

We computed the changes in the scores as differences from Time1 to Time2 in the experimental group. To summarise the variability of these new variables and to reduce their dimensionality, we used exploratory factor analysis. The results showed two weakly correlated components (r = .16) that globally explained 52% of the variance. The first component concerned changes in the death measures; the more relevant variables (i.e., with higher factor loadings) were the fear of death, the attitude toward care of the dying, the avoidance of death and the death representation. The second component concerned changes in self-efficacy and career commitment (Table 3).

**Table 3:** Factor loadings of change scores on each component

Variables	Component 1	Component 2
Change scores on TDRS	0.56	0.09
Change scores on Fear of Death	0.76	0.03
Change scores on Death Avoidance	0.67	0.24
Change scores on FATCOD	0.75	-0.28
Change scores on CCS	0.04	0.83
Change scores on CSE	0.01	0.62

**Fig. 1:**
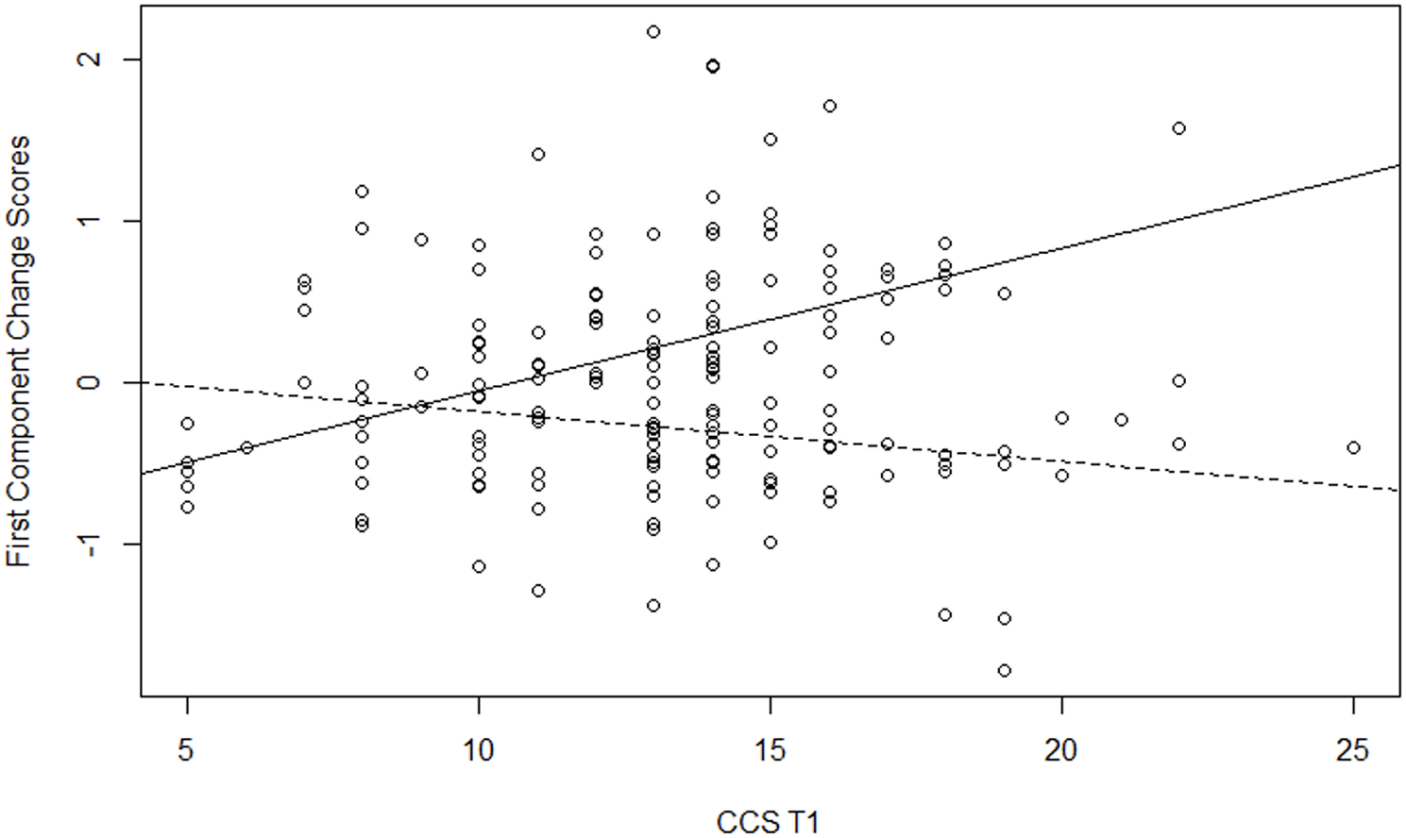
Moderation of TDRS in the impact of CCS on the First Component Change Scores. Legend: dotted line for students with low scores on TDRS, continuous line for students with high scores on TDRS

**Fig. 2:**
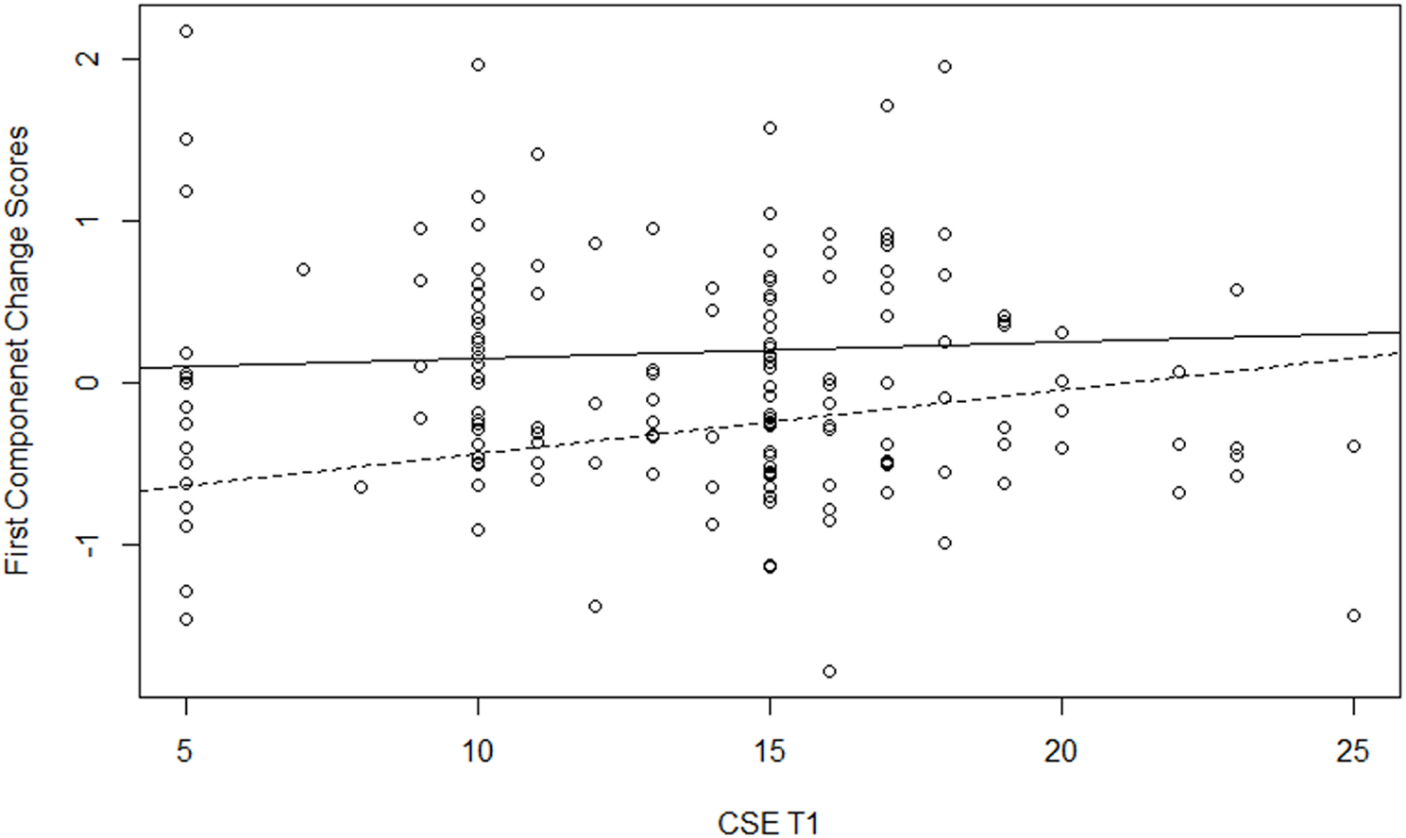
Moderation of TDRS in the impact of CSE on the First Component Change Scores. Legend: dotted line for students with low scores on TDRS, continuous line for students with high scores on TDRS

**Fig. 3:**
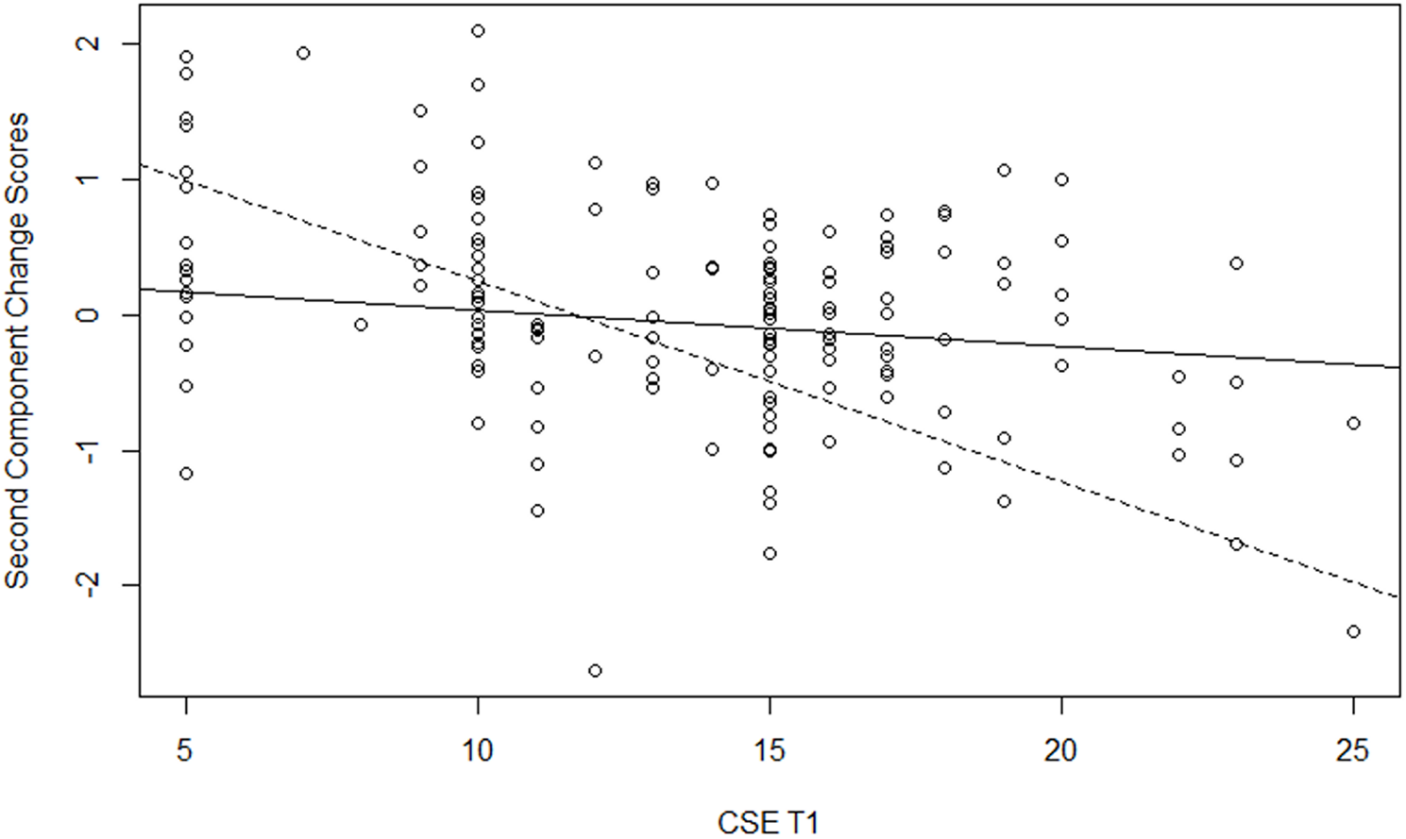
Moderation of FATCOD in the impact of CSE on the Second Component Change Scores. Legend: dotted line for students with low scores on FATCOD, continuous line for students with high scores on FATCOD

TDRS = Testoni Death Representation Scale; FATCOD = Frommelt Attitude Toward Care of the Dying Scale; CCS = Career Commitment Scale; CSE = Creative Self-Efficacy Scale;

To explain the variability of the two components, we used hierarchical regression analysis, adding predictors in three steps. We included as control variables the country, age, gender, religious level, care experience and loss experience in the first step; the variables at Time1 in the second step; and the significant interactions of the variables at Time1 with the moderator in the last step. We fitted several models include as moderator in turn each variable at Time 1. The results of the best model for each component are reported in Table 4. Model 1, with only control variables, explained a significant quote of variance of the first component (R-squared = 19%) but not of the second component (R-squared = 9%). In particular, there were differences between countries, with greater positive changes in Italy than in the other countries for the first component, whereas for the second component, the previous experience as a formal caregiver had a significant negative impact (i.e., those who had this experience changed less). Moreover, in Model 2, after controlling for the measures at Time1, the religious level had a positive impact on both components, with greater positive changes for those who were more religious; and in Model 3, after controlling for the measures at Time1 and their interactions with death representation, gender had a positive impact only for the first component, with greater positive changes for women. Model 2, with measures at Time1 added to control the variables, showed significant variance (39% for the first component and 25% for the second component), with the initial scores having a positive contribution to the variables related to the representation of death, avoidance of death and career commitment for the first component, and a negative contribution to creative self-efficacy for the second component. Model 3 is the final model for the first component, with a significant quote of variance explained by the moderation of the death representation (Delta R-squared = 6%, p = .009); and Model 4 is the final model for the second component, with a quasi-significant quote of variance explained by the moderation of the attitudes towards the care of the dying (Delta R-squared = 5%, p = .056). Significant moderation of death representation in the impact of career commitment on the first component was found: the positive changes at the end of the course depended on career commitment only for those with high death representation scores (B = 0.09, SE = 0.04, p = .044), while they were not influenced by career commitment for those with low death representation scores (B = -0.03, SE = 0.02, p = .175). The positive sign of the slope parameter indicates that there were more positive changes at the end of the course, with a higher initial career commitment of students with a death representation close to annihilation (continued line in the plot, Fig. 1). Moreover, significant moderation of death representation in the impact of creative self-efficacy on the first component was found: the positive changes at the end of the course depended on creative self-efficacy only for those with low death representation scores (B = 0.04, SE = 0.02, p = .049), while they were not influenced by creative self-efficacy for those with high death representation scores (B = 0.01, SE = 0.03, p = .764). The positive sign of the slope parameter indicates that there were more positive changes at the end of the course for the students with a death representation farther from annihilation and with higher initial creative self-efficacy (dotted line in the plot, Fig. 2). Finally, significant moderation of attitudes towards the care of the dying by the impact of creative self-efficacy on the second component was found: positive changes at the end of the course depended on creative self-efficacy only for those with low attitude toward care of the dying scores (B = -0.15, SE = 0.04, p = .004), while they were not influenced by creative self-efficacy for those with high attitude toward care of the dying scores (B = -0.03, SE = 0.04, p = .474). The negative sign of the slope parameter indicates that there were more positive changes at the end of the course among students with a low attitude towards the care of the dying and had lower initial creative self-efficacy (dotted line in the plot, Fig. 3).

**Table 4:** Regression models for the two Components of Change Sores observed in experimental group (N = 165)

Predictor	First Component						Second Component					
	Model 1		Model 2		Model 3 (moderator TDRS)		Model 1		Model 2		Model 4 (moderator FATCOD)	
	B	SE	B	SE	B	SE	B	SE	B	SE	B	SE
Control variables												
Country_D1 (Austria = 1; Italy = 0)	-0.48**	0.14	-0.28*	0.13	-0.23	0.13	-0.23	0.17	-0.28	0.16	-0.25	0.16
Country_D2 (Romania = 1; Italy = 0)	-0.47*	0.18	-0.43*	0.17	-0.45**	0.16	-0.35	0.21	-0.25	0.21	-0.23	0.20
Country_D3 (Israel = 1; Italy = 0)	-0.60**	0.18	-0.42*	0.17	-0.42*	0.17	-0.33	0.22	-0.18	0.21	-0.17	0.21
Country_D4 (Poland = 1; Italy = 0)	-0.66***	0.16	-0.34*	0.16	-0.35*	0.15	0.06	0.19	0.08	0.19	0.09	0.19
Age	0.00	0.01	0.00	0.01	0.00	0.01	0.02	0.01	0.02	0.01	0.01	0.01
Gender (Female = 1; Male = 0)	0.26	0.14	0.25	0.13	0.32*	0.13	-0.09	0.17	-0.02	0.16	-0.01	0.16
Religious level	0.07	0.06	0.13*	0.06	0.15*	0.06	0.12	0.07	0.15*	0.07	0.15	0.08
Care Experience (Yes = 1, No = 0)	-0.24	0.20	-0.22	0.18	-0.18	0.18	-0.524*	0.24	-0.34	0.23	-0.26	0.23
Loss Esperience (Yes = 1; No = 0)	-0.10	0.11	0.00	0.10	0.05	0.10	0.20	0.13	0.11	0.12	0.08	0.12
Measures at T1												
TDRS			0.03**	0.01	0.01	0.14			0.01	0.01	0.16	0.13
Fear of Death			0.12	0.07	-0.18	0.22			0.02	0.09	-2.20	1.20
Avoidance of Death			0.19**	0.07	0.16	0.25			-0.02	0.09	0.93	1.05
FATCOD			-0.22	0.18	0.29	0.66			0.27	0.23	-0.83	1.54
CCS			0.03*	0.02	-0.13**	0.05			-0.03	0.02	0.16	0.22
CSE			0.00	0.01	0.08*	0.04			-0.06***	0.01	-0.44*	0.16
Interactions with moderator												
TDRS x moderator											-0.04	0.03
Fear of Death x moderator					0.01	0.01					0.55	0.30
Avoidance of Death x moderator					0.00	0.01					-0.23	0.27
FATCOD x moderator					-0.02	0.03						
CCS x moderator					0.01***	0.002					-0.05	0.06
CSE x moderator					-0.004*	0.002					0.10*	0.04
R-square	19%*		39%*		45%*		9%		25%*		30%*	

TDRS = Testoni Death Representation Scale; FATCOD = Frommelt Attitude Toward Care of the Dying Scale; CCS = Career Commitment Scale; CSE = Creative Self-Efficacy Scale

## Discussion

Based on the results, it is possible to say that the DeEd course to introduce PC to university students from the five European countries (Austria, Israel, Italy, Poland and Romania) who participated in the trial confirmed our basic hypothesis: it is possible to realise DeEd courses without creating or increasing death anxiety and to motivate students to work in PC in the future. In accordance with the Lancet Commission on the Value of Death [46] and with what has already been emphasised in literature [13, 31], it is possible to bring the issues of death and dying back into the real lives of people and thus, also of students. It would also be useful to introduce PC in depth to students who could potentially work in the healthcare field in the future. Contrary to common sense opinions, it is possible to address these issues seriously and competently without necessarily causing discomfort and despondency in students. Moreover, through these courses, it is possible to increase the predisposition to work in the PC field.

The changes over time in the experimental and control groups highlight how the view of death as annihilation is correlated to the fear of death and to the need to avoid thoughts concerning dying. This attitude is thus inversely correlated to the acceptance of death and even more strongly, to carer commitment, self-efficacy and the attitude towards caring for dying people. As considered by the Lancet Commission for PC [47], ignorance of PC is an obstacle to realising the universal human right to reduce suffering whenever possible. In line with this, the initial result of DE4PP confirms the need in DeED courses to consider the issue of personal representations of death because very often, they are confused and unconscious, but they influence attitudes towards finitude, unconsciously inhibiting the willingness to help those who are facing death.

These findings are in line with our exploratory factor analysis of the changes in the score, which highlight two factors that must be considered between pre- and post-DeEd interventions: Component 1, describing changes between representations of death, fear and avoidance of death; and Component 2, concerning self-efficacy and career commitment. As illustrated by previous research [40], the belief that death means annihilation in totality causes avoidance of this topic and reduces resilience in the face of illness. However, people are not always fully aware of this reaction of theirs and therefore, it is necessary to allow them to face the topic and manage it to reduce their anxiety and avoidance [48]. This awareness is particularly useful for potential healthcare professionals. Thus, the two components confirm the existence of this relationship on which it is possible to work with death education courses: self-competence with respect to fear of death and representations of death, and the acquisition of skills to improve communication and relationship skills in order to work with dying patients and their relatives in PC teams.

In line with this kind of reflection, as indicated by the hierarchical regression results regarding the control variables, the Italian students more notably reduced their representation of death as annihilation, fear and avoidance of death than did the students in the other countries, even though they had experienced caring for sick and dying people. This first result is in line with the results in literature that show that DeEd courses aimed at dealing with representations of death did not increase the students’ anxiety levels but helped them to better manage these issues [14–18, 49]. Moreover, as also already discussed extensively in literature [31], more religious or spiritual students are more likely to cope better with death and dying; and also in this course, they proved to be the ones who gained the most, further decreasing their representation of death as annihilation, death anxiety and avoidance, and increasing their levels of self-efficacy and willingness to work in PC. This effect was achieved particularly among women, who, again as already discussed in literature, seemed to favour religious attitudes more [50].

The second regression model results regarding the study variables showed an interesting change: stronger support for including PC DeEd in university courses for future health professionals, without the risk of creating discomfort or rejection of this professional possibility. In fact, it appears that even those who most represented death as annihilation significantly increased their willingness and vocation to work in the field of PC. This means that despite their pessimistic view of death, having had the opportunity to deal with the subject and consider the PC topics, as well as having learned techniques to improve their interpersonal and communication skills, enabled them to imagine being able to work with the dying and their relatives. Literature has already highlighted that PC competence facilitates familiarisation with issues of death and dying as well as the ability to work in this area, thereby enhancing interpersonal skills [18, 51–53]. On the other hand, those who represented death as a passage gained, at the end of the course, a greater level of confidence in their own abilities to find new and creative solutions for interventions in end-of-life situations.

The final regression model results for the first factor confirmed that the difference between the two forms of death representation (annihilation or passage), in light of what TMT [9, 40], according to which the ability to think of oneself beyond death helps one to better manage the fear of death, can be interpreted in this way: those who represented death as annihilation enhanced their ability to manage their fear of death when they imagined being able to work in PC (Fig. 1); those who instead represented death as a passage had less trouble thinking about working in this area and were thus able to go one step further [i.e., after a familiarisation process with PC, they felt more able to manage their fear of death by being more creative and effective in this area (Fig. 2)]. This result confirms the importance of using psychodrama and creative arts methodologies to approach PC and death-related topics. Indeed, as research has already amply demonstrated, the use of these therapeutic techniques with an enhanced ability to empathise with those suffering and to communicate [54–57] helps to increase levels of creativity, problem solving skills and the promotion of change [18, 58–61].

On the other hand, the results of the final regression model for the second factor showed that those who initially felt less self-confident and were therefore less inclined to work in the field of PC later had difficulty feeling more able to find new and creative solutions in the field of PC (Fig. 3). This means that it is necessary, within DeEd paths for PC, to verify the feeling of powerlessness with respect to the possibility of working in PC and to propose further activities that reduce this feeling. However, despite this specific difficulty, the DE4PP results seem to be extremely encouraging in suggesting that these kinds of courses aimed at university students cause neither an increase in defensive and negative attitudes towards death and dying nor a distancing from the possibility of choosing PC as a field of work in the future.

## Study limitations and suggestions for future research

With regard to the limitations of the DE4PP intervention, not all partners addressed the topic of representations of death as annihilation or passing away systematically but only tangentially. The second limitation is that, as a result of Model 4, no further support work was undertaken for the students, who showed that they felt more uncertain about these issues and thus, insecure about the possibility of being able to provide creative solutions in possible PC work.

As for this study, the limitations especially concerned the different number of participants in the different countries. This is because the numbers of students in the different degree courses also differed. In addition, all the students were from psychology, not involving students from other health professions such as medical or nursing students. Future research should increase the number of participants, for greater promotion of these initiatives among potential future healthcare professionals, also involving medical and nursing students to assess the impact of the course on them.

## Conclusion

As internationally discussed, it would be necessary to include systematic PC DeEd courses in degree courses that train potential healthcare professionals.

DE4PP has shown that it would be important to implement PC DeEd courses because they can help healthcare students to tool to better cope with death related difficulties. In addition, the present study shows that psychodrama and creative arts therapies can be useful tools to be employed in PC DeEd interventions. Both face-to-face and distance learning activities supported the students’ familiarisation with the issues related to death and dying, which may have increased their propensity to turn to this type of field in their future profession.

## Data Availability

The data that support the findings of this study are openly available in Research Data Unipd at 10.25430/researchdata.cab.unipd.it.00000790. Data may be made available upon reasonable request from the corresponding authors.
